# Strand-specific detection of cell-associated sense and antisense HIV-1 RNAs in splenocytes and PBMC from PLWH

**DOI:** 10.1186/s12977-025-00670-5

**Published:** 2025-12-24

**Authors:** Laetitia Waast, Aurélien Geronimi, Adeline Mélard, Suzanne Figueiredo, Matthieu Maisch, Jean-Paul Viard, Véronique Avettand-Fenoel, Jacques Dutrieux, Claudine Pique

**Affiliations:** 1 Université Paris Cité, CNRS, INSERM, Institut Cochin, F-75014 Paris, France; 2 Université d’Orléans, LI²RSO and CHU d’Orléans, Orléans, France; 3 Service d’Immuno-Infectiologie, Ecole de Médecine, Hôpital Hôtel-Dieu, Assistance Publique-Hôpitaux de Paris and Université Paris Cité, Paris, France; 4 Dynavir Consortium, CNRS GDR 2194, Paris, France

**Keywords:** Lentivirus, Cell-associated RNA, Antisense, RTddPCR, Subtype

## Abstract

**Background:**

Variation in the level of cell-associated HIV-1 RNA is an important parameter followed in clinical trials focusing on HIV-1 infection, latency or reactivation. In addition to sense products, HIV-1 also expresses antisense products that can modulate HIV-1 replication, either positively through the antisense protein ASP or negatively through repressive noncoding antisense RNAs. Therefore, quantification of both sense and antisense HIV-1 products could provide key information for monitoring the dynamics of viral replication in vivo. While the ASP protein is difficult to detect even in vitro, antisense RNAs can be detected both in vitro and in vivo. The aim of this study was therefore to establish protocols for the specific quantification of sense and antisense transcription that can be applied in clinical studies. To this end, we developed strand-specific RT-PCR protocols allowing us to quantify individually sense and antisense RNAs in PLWH with B and non-B viruses.

**Results:**

We show that the two RTqPCR protocols can quantify standard HIV-1 sequences with good analytical parameters. We also demonstrate the strand specificity of the two protocols by showing that RNAs of the other orientation do not contaminate RNAs of one orientation during the PCR step and that the sense and antisense RT-qPCR protocols detect distinct populations of HIV-1 RNAs. We then compared the sensitivity of RT-qPCR and RT-ddPCR to quantify HIV-1 cell-associated RNAs and found that RT-ddPCR results in lower inter-sample variation or higher levels of detection than RT-qPCR. Finally, we show that the RT-ddPCR protocols efficiently quantify cell-associated HIV-1 sense and antisense RNAs not only in HIV-1-infected primary CD4 + T cells but also in spleen and blood samples from untreated HIV-1-infected individuals.

**Conclusions:**

This study demonstrates that HIV-1 antisense RNAs are expressed in spleen and blood of untreated HIV-1-infected individuals, of at least B and CRF02 subtypes, and that the level of antisense transcription can be significant and even predominant, as compared to sense transcription. These data, along with the protocols we described, will enable a more thorough analysis of HIV-1 sense and antisense expression dynamics in vivo, which could pave the way for novel strategies to control HIV-1 infection.

**Supplementary Information:**

The online version contains supplementary material available at 10.1186/s12977-025-00670-5.

## Introduction

Retroviruses integrate their reverse-transcribed genomes into host cell chromatin to generate the provirus, which contains the viral genes flanked by two long terminal repeats (5’ or 3’LTR) carrying the regulatory elements that control viral transcription. It was long assumed that 5’LTR-driven RNA synthesis from the positive strand was the only type of retroviral provirus transcription. However, the possibility that retroviruses might exhibit antisense transcription was first suggested in 1988 by a computer analysis revealing a putative antisense open reading frame (ORF) in the HIV-1 genome [1]. The production of antisense viral RNAs was later demonstrated, first in human T-cell leukemia virus type 1 (HTLV-1) [2, 3] and subsequently, in human immunodeficiency virus type 1 (HIV-1) [4–6].

In the case of HIV-1, the antisense ORF overlaps with the sense Rev-Responsive Element positioned in the *env* gene [7]. The antisense ORF encodes for the AntiSense Protein, ASP, which localizes to cellular membranes and induces autophagy [8]. Furthermore, ASP colocalizes with viral envelope proteins at the plasma membrane and is incorporated into the virion envelope [9]. In addition, a recent study demonstrates that ASP enhances HIV-1 entry into CD4 + T cells [10]. Antibodies and CD8 + T lymphocytes directed against ASP have been detected in people living with HIV-1 (PLWH), indicating that antisense expression occurs during chronic infection [5, 11–13]. This, along with the fact that a complete ASP ORF is associated with faster disease progression [14], suggests positive roles of ASP in HIV-1 replication and pathogenesis. However, the situation is more complicated since antisense products also negatively influence HIV-1 replication by operating as non-coding RNA that can repress the 5’LTR [15–19]. These noncoding antisense HIV-1 transcripts carry inefficient polyA signals and are predominantly found in the nucleus [20]. The mechanistic basis of repression is the binding of these RNAs to the 5’LTR, followed by the recruitment of epigenetic repressors such as the PRC2 complex or HDAC [16, 17].

Given their potential roles in HIV-1 replication, the in vivo expression of HIV-1 antisense products could be particularly important not only during chronic or latent infection but also in the context of anti-HIV-1 therapies. *Via* the cooperation with the envelope glycoproteins, ASP could facilitate virus entry, which is a mechanism that could be exploited to develop novel antiviral drugs interfering with virus transmission [9]. Moreover, activation of the autophagic pathway by ASP may affect cell protein degradation as well as antigen presentation [8, 21]. On the other side, the demonstration that antisense RNAs induce 5’LTR repression and block LTR reactivation [15] suggests that antisense RNAs could play a key role in the establishment or maintenance of HIV-1 latency [22]. Antisense HIV-1 RNAs could therefore be part of therapeutic strategies aiming at either reversing (shock-and-kill) or reinforcing (block-and-lock) latency [23].

Detection of the ASP protein in vitro has been shown to be very challenging [21] and is therefore unlikely to be achievable in PLWH. In contrast, HIV-1 antisense RNAs have been detected in PBMCs from PLWH. First, Zapata et al. developed a tag-based strand-specific RTqPCR method to detect HIV-1 antisense RNAs in 3 PLWH on antiretroviral therapy (ART), with very low expression levels of 10 to 30 copies/million CD4 + T cells [16]. Then, Mancarella et al. showed expression of HIV-1 antisense RNAs in PLWH, but only after ex vivo PBMC restimulation, using an enrichment method based on RT product purification [24]. Recently, also Capoferri et al. used a tag-based RT but coupled to droplet digital PCR (ddPCR) to quantify antisense RNAs expression in unstimulated PBMCs from 3 PLWH on ART and 5 untreated PLWH, and described a level below 20 absolute copies/100 HIV-1-infected cells [25]. Of note, these studies focused on HIV-1 B subtype, considered to be predominant in western countries. However, several reports indicate that infections caused by circulating recombinant forms (CRFs), such as CRF02 and CRF06, are increasing regularly in these regions [26].

The level of cell-associated HIV-1 RNA is one of the viral parameters used to monitor HIV-1 replication, viral reservoir formation or viral reactivation [27, 28]. To date, only sense RNAs are quantified, although parallel quantification of antisense products could provide new insights into HIV-1 replication dynamics in vivo. Given the apparently low level of HIV-1 antisense RNA production in vivo, the quantification methods must be highly sensitive. Furthermore, since antisense RNAs overlap with sense RNAs, strand-specific detection is required for excluding contamination of one orientation transcript by the other. In addition, viral diversity must be considered for targeting the most prevalent viral subtypes in a given region [26]. In this context, the aim of this study was to develop optimized RT-PCR protocols that allow for the sensitive, strand-specific quantification of HIV-1 transcripts, while accounting for viral diversity. Here, we describe the protocols, their validation and their application to the quantification of HIV-1 sense and antisense transcripts in cell lines, primary CD4 + T cells as well as clinical samples from PLWH of B and CRF-02 subtypes.

## Methods

### Cells and clinical samples

The latently-infected HIV-1 T-cell lines ACH-2 and J1.1 or uninfected Jurkat T cells were kindly provided by S. Emiliani (Institut Cochin, Paris). Blood from a normal donor was obtained from the Etablissement Français du Sang under convention number 18/EFS/30. Primary CD4 + T cells were purified by negative selection using the EasySep Negative Human CD4 Kit (Stem Cell technologies) and were cultivated for 5 days in the presence of IL-7 (10ng/mL, Miltenyi Biotec) before infection. Primary spleen cells were obtained from three untreated PLWH followed between 1992 and 1996 who underwent splenectomy to treat thrombocytopenia [29]. These samples were classified as surgical waste and, therefore, neither ethical approval nor informed consent was required for their use under French law at that time.

PBMCs from three untreated PLWHs were purified from 10 ml blood samples through Ficoll gradient centrifugation and cryopreserved immediately in RLT buffer then stored at -80 °C. The study of sense and antisense HIV-1 RNAs production in PLWH was approved by the ethic committee (CPP Ouest III, decision number #21.12.86 /SI RIPH2G 21.03737.000039, December 22 2021) and all participants gave their written consent. HIV-RNA loads were quantified with the Alinity m HIV-1 assay (Abbott) according to the manufacturer instructions. HIV subtypes were determined by sequencing Pol sequences from HIV RNA prepared from plasma and analysis using the HIV resistance database from Stanford University (http://dbpartners.stanford.edu:8080/RegaSubtyping/stanford-hiv/typingtool/).

### In vitro HIV-1 infection

The laboratory strain NL4.3 harboring a VSV-G pseudo-typed envelope was produced and concentrated by ultracentrifugation by co-transfection of pNL4.3 (NIH AIDS Reagent) and of a pVSV-G (Addgene) encoding plasmids. Viral concentration was estimated using Enzyme-Linked Immunosorbent Assay (ELISA) of p24 protein (Lenti-X p24 Rapid Titer kit, Takara Bio), following the manufacturer’s protocol. The infectious dose 50 (ID50) of each virus was determined by serial dilution of the viruses on reporter cell line HeLa TzmBL (NIH AIDS reagent program) and β-galactosidase activity was determined 24 h post-infection using β-galactosidase enzyme assay (Promega) according to the manufacturer’s protocol. Primary CD4 + cells were left unstimulated in the presence of 10ng/mL IL-7 (Miltenyi Biotec) to ensure cell viability. Cells were infected with a viral concentration of 1 ID_50_ during 2 h in a serum free medium. Cells were then washed by centrifugation at 600 g for 5 min and resuspended in complete medium (RPMI 1640 supplemented with 10% FBS and 1% Penicillin/Streptomycin) and cultured in presence of 10ng/mL of IL-7.

### Preparation of total and fractionated RNAs

Total RNA was isolated using the Nucleospin Macherey Nagel miRNA extraction kit. This kit eliminated small RNAs (< 200 b), reducing non-specific priming during the RT step. The preparation of cytoplasmic and nuclear RNAs was performed using the RNA Subcellular Isolation Kit from Active motif, according to the manufacturer’s recommendations. Briefly, cells were lysed in Buffer x plus DTT and cell nuclei and cytoplasmic fractions were separated by centrifugation. Supernatants containing the cytoplasm and pellets containing the nuclei were then treated separately on columns to extract RNA (> 75 nucleotides). RNAs were eluted using 40 to 50 µL of RNase-free water and used immediately or stored at -80 °C.

### PCR validations on standard DNAs

**Fig. 1:**
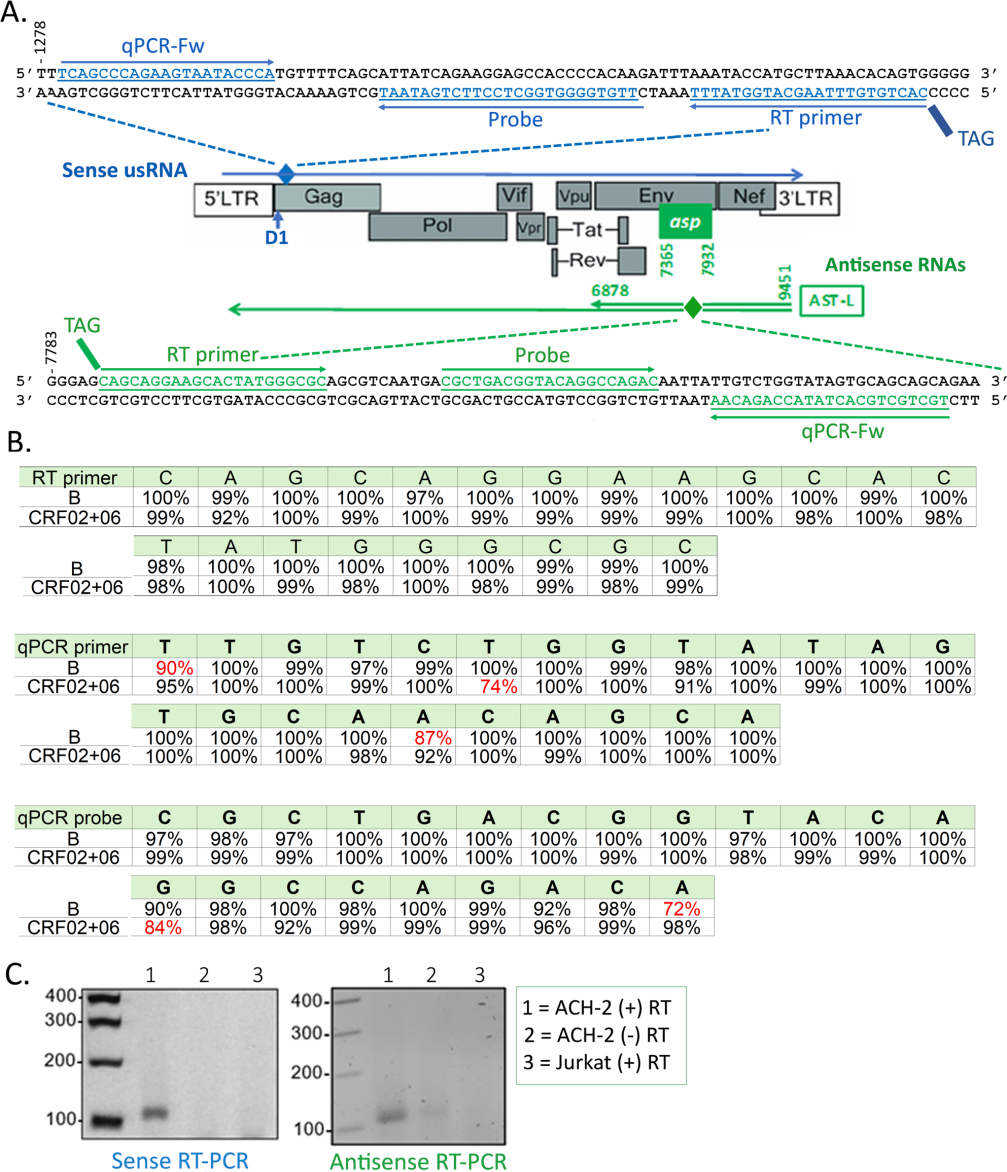
Design of the orientation-specific RT-qPCRs. **A** Schematic representation of the HIV genome showing the sense unspliced RNA (usRNA) in blue and the antisense RNAs in green. The position of the predominant species of antisense RNA, ASP-L, is shown. The positions and orientations of the sense and antisense probes and primers are indicated in the upper or lower part of the figure. **B** Conservation of the antisense probe and primers was assessed using the 2020 HIV Compendium among 1,072 B and 133 CRF-02 + CRF-06 sequences. Positions with degree of variability ≤ 90% are in indicated in red. **C** Gel analysis of the PCR products amplified with either the sense or the antisense RT-PCR protocol using total RNAs extracted from the HIV-1 latently-infected ACH-2 T cells or uninfected Jurkat T cells

Validations of the PCRs were performed using double strand DNA fragments (dsDNA) (Strings DNA fragment, Invitrogen) corresponding to the sense HIV-1 unspliced (us)RNA or the total antisense HIV-1 RNAs amplicon, from the tag sequence to the forward primer sequence (see Fig. 1A). For determining inter-assay variability, ten-fold serial dilutions of each dsDNA from 10^7^ copies to 1 copy were amplified in 6 (usRNA) or 9 (antisense RNA) independent PCR reactions, performed in triplicates. For intra-assay variability determination, the same PCR reaction was quantified 10 time in triplicates. The coefficient of variation (CV) for each number of copies was calculated as followed: (SD/mean Ct)x100. PCR efficiencies were calculated using the “ThermoFisher qPCR efficiency calculator” from the slope of the regression curve of the inter-variability experiments. The limit of blank (LoB) was determined by parallel quantification of 10 PCR reactions containing only the mix/enzyme solution (no template control). Limit of detection (LoD) was determined as the lowest dilution for which at least 95% of the replicates were positive and limit of quantification (LoQ) as the lowest dilution for which 100% of the replicates were positive.

### cDNA synthesis

To achieve sense-specific detection, we used the strand-specific RT-PCR method based on a RT primer composed of a viral sequence fused to a neutral sequence (tag: 5’ GATCTAGGA GCTACGAGTCCAA 3’), the tag sequence serving subsequently as reverse primer during the PCR reactions. The tagged-primers were 5’ GATCTAGGA GCTACGAGTCCAACACTGTGTTTAGCATGGTATTT3’ for the sense RT and 5’ GATCTAGGA GCTACGAGTCCAACAGCAGGAAGCACTATGGGCGC3’ for the antisense RT. RT reactions were carried out in a total volume of 50 µL with 250 to 1000 ng of total RNA using the kit “Maxima First Strand cDNA Synthesis Kit with dsDNAse” (ThermoFisher Scientific). Treatment with dsDNase for 5 min at 37 °C was performed to eliminate residual genomic DNA. Then 1 µL of dNTP (10 nM) and 1 pM of the tagged-RT primer (custom DNA oligo, HPSF quality, Eurofins genomics) were added before addition of the RT enzyme and buffer (maxima H minus, Thermofisher). After a 30 min incubation at 55 °C (sense RT) or 65 °C (antisense RT), the RT enzyme was inactivated at 85 °C for 5 min and RT products were used immediately for PCR or stored at -20 °C.

### Gel analysis of PCR products

End-point PCR were performed with 250 ng of cDNA mixed to 0,5µL of dNTP (10 mM), 1µL of the sense or antisense PCR primers (10 pmol/µL, see Fig. 1A), 0,125 µL of polymerase enzyme (OneTaq DNA Polymerase, NEB), 5µL of buffer (One Taq Standard Reaction Buffer, NEB) and RNA-free water to reach a final volume of 25µL. The following PCR program was used: first denaturation step at 95 °C for 10 min, second denaturation step at 95 °C for 10s, primer hybridization at 60 °C for 30s, elongation at 68 °C for 1 min, the three last steps being repeated 50 times. Migration of the PCR products was performed in a 2% agarose gel (Agarose medium-low EEO, Thermofisher Scientific) in TBE 0,5X containing ethidium bromide EtBr (250 ng/mL). Fragments visualization was achieved by UV exposition using a FUSION FX camera (Vilber).

### Quantitative PCR

The sense usRNA and total antisense HIV-1 RNAs were quantified by RTqPCR using the Taqman method. The sequences of the primers and probes (Fig. 1A) were blasted to the NCBI GenBank to ensure no complementarity with the human genome. Following RT, two µL of the reaction was used in the qPCR (45 cycles, denaturation 95 °C for 10 s, hybridization 60 °C for 30s, elongation at 68 °C for70 sec, the three last steps being repeated 50 times) with 2 pmol/reaction of probes and primers in a volume of 10µL and the PCR enzyme SSoADV universal probes supermix (Bio-RAD ref 1725281). A classical oligodT RT-qPCR was performed on the same RNA samples to quantify the levels of housekeeping genes HPRT and EEF1G using primers and probes from Life Technology (ref 4331182.)

### RTddPCR

RTddPCR were done using the same primers and probes as for RTqPCR. Reactions were performed in 20µL including 4µL of sample. A premix containing the ddPCR Supermix for Probes (no dUTP), 10pmol of primers and 5pmol of probes was made for 8 points. The micro-droplets were generated according to the manufacturer’s recommendations (QX200 Droplet Generator, Biorad) and were transferred to a sealed 96-well plate before the end-point PCR (50 cycles, denaturation at 95 °C for 30 s, hybridization and elongation at 60 °C for 1 min) before analyses using the QX200 Droplet reader (Biorad). For each strip of 8 points, one negative control was included to set the quantification threshold required to identify positive droplets. The total number of copies was determined by summing the number of positive droplets obtained for each well divided by the total reaction volume followed by normalization to the quantity of RNA used in the reaction.

### Editing of the manuscript

Parts of the manuscript were edited using the DeepL Write correction IA web site.

## Results

### Design of the strand-specific RT-qPCRs

In cases where viruses exhibit transcription from both the plus and minus strands, separate detection of each RNA population requires a strand-specific RT-PCR method [30]. Indeed, such methods were used to demonstrate the production of antisense HIV-1 RNAs in vitro and in vivo [16, 18]. Stand-specificity is achieved by fusing in the RT primer, a non-viral sequence (Tag) to a viral sequence, the tag being subsequently used as one of the PCR primers so that only cDNAs tagged during the RT step would be amplified during the PCR. The unspliced sense RNA (usRNA) was amplified *via* primers and probes that amplify a conserved region of *gag* positioned downstream of the D1 donor splicing site [31] (Fig. 1A). For antisense RNAs, we initially focused on the region targeted in Zapata et al. (nuc 7489–7554) for detecting antisense RNAs in PLWH [16]. However, inspection of HIV-1 sequences (HIV Compendium 2020) revealed variability within the PCR primer and/or probe sequences used in this study, as well as in the recent study by Capoferri et al. [16, 25]. (Fig. S1). We therefore searched for a more conserved region and selected the nuc 7787–7869 one, in which we could identify primers and the probe exhibiting high degrees of conservation not only within clade B strains but also for the CRF-02 and CRF-06 recombinants (Fig. 1B). The sizes and purities of the amplified products were then analyzed using total RNAs from the latent T-cell line ACH-2 cells and from non-infected Jurkat T cells (Fig. 1C). The expected fragment sizes were observed for the sense (113 bp) and antisense (103 bp) RT-qPCRs (Fig. 1C, lanes 1). Of note, sequencing of the PCR products indicated that the amplified products presented the expected sequences (not shown). No amplification product was found with total RNAs from Jurkat T cells (Fig. 1C, lanes 3) and no (sense) or only traces (antisense) of amplification products were found with ACH-2 RNA treated in the absence of the RT enzyme (Fig. 1C, lanes 2).

### Analytical parameters of the sense and antisense RT-qPCRs

**Fig. 2:**
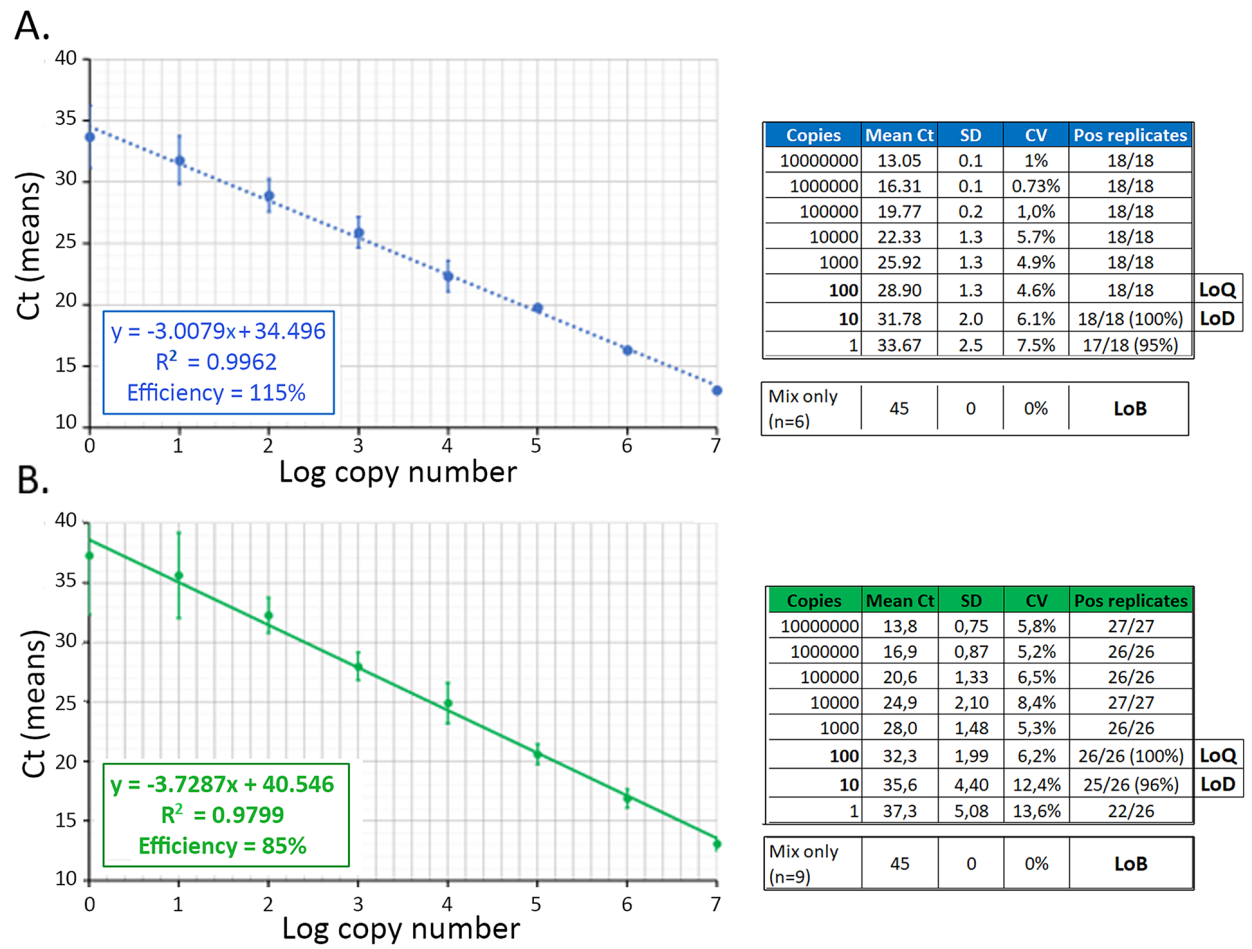
Analytical parameters of the sense and antisense PCRs. **A** Standard curve of the sense PCR and **B** Standard curve of the antisense PCR, each determined using 10-fold dilutions of double strand oligonucleotides. Inter-assay variability was assessed by parallel quantifications in duplicates of 6 (sense PCR) or 9 (antisense PCR) independent dilutions. The PCR efficiencies were calculated from the slope of the standard curve using the “ThermoFisher qPCR efficiency calculator”. The right tables show the mean Ct values as well as the standard deviation and coefficients of variation for each number of copies. The limit of detection (LoD), limit of quantification (LoQ) and limit of blank (LoB) determined as indicated in the Method section are also indicated in the tables

The analytical parameters of the RTqPCRs were determined using double-stranded DNA (dsDNA) oligonucleotides corresponding to the sense or antisense amplicon. Serial 10-fold dilutions of the dsDNA were used to determine the intra-assay and inter-assay variations. The coefficients of variation (CV) for intra-assay variabilities were less than 5% for both the sense and antisense PCRs (Fig. S2). Regarding inter-assay variabilities, CV below 8% and 14% were found for the sense and antisense RTqPCR, respectively (Fig. 2A and B, right tables). In addition, the two PCRs exhibited satisfactory efficiency values of 115% (sense qPCR) and 85% (antisense qPCR) and linearity values (R^2^) of 99.6 (sense qPCR) and 98 (antisense qPCR) (Fig. 2A and B, left panels). The limit of blank (LoB) was 45 cycles for both the sense and antisense RTqPCRs (Fig. 2A and B, right tables). Lastly, the limit of detection (LoD) was found to be 1 (sense qPCR) or 10 (antisense qPCR) copies per reaction, and the limit of quantification (LoQ) determined as 10 (sense qPCR) and 100 (antisense qPCR) copies per reaction (Fig. 2A and B, right tables).

Together with the above findings, these data indicate that the sense and antisense RTqPCRs quantify single products of the expected sequences, display correct intra- and inter-assay variabilities, linearities and efficiencies, and detect as low as 10 viral copies per reaction. These results validated these protocols for further quantifications of cellular-associated HIV-1 RNAs.

### Validation of the sense and antisense RTqPCRs using cellular RNA

**Fig. 3:**
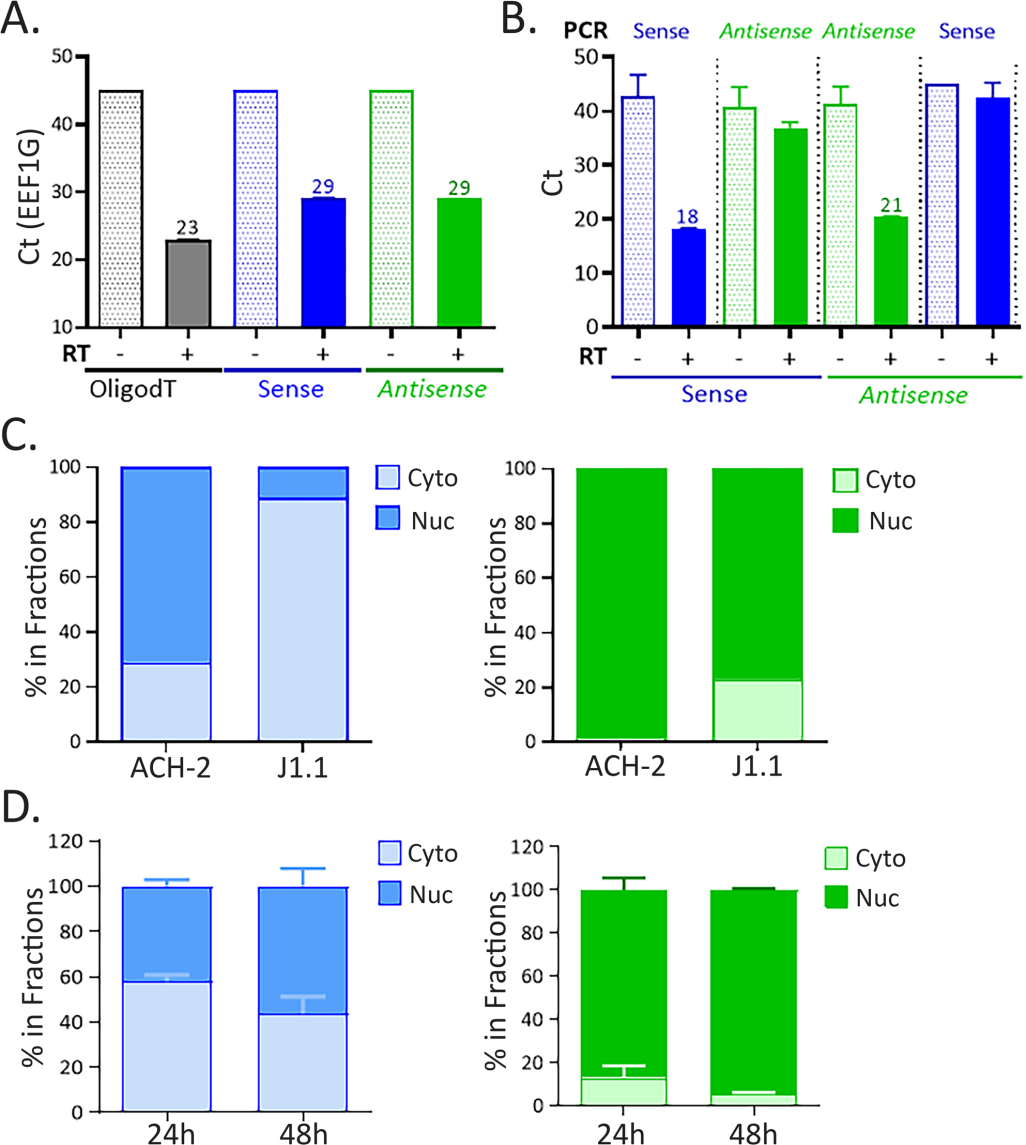
Validations of the sense and antisense RTqPCRs. **A** Quantification of the EEF1G RNA following RT reactions performed with oligodT, HIV-1 sense or HIV-1 antisense RT primer using ACH-2 total RNAs. **B** Quantification of the HIV-1 RNAs using either homologous (sense/sense or antisense/antisense) or opposite (sense/antisense) RT and PCR primers using ACH-2 total RNA. **C**, **D**. Intracellular distribution of HIV-1 RNA quantified by either the sense or antisense RTqPCR protocol using fractionated RNAs from (**C**) ACH-2 or J1.1 T cells or (**D**) Jurkat T cells infected with NL4.3 viruses for 24–48 h. The percentage of RNAs in each fraction was calculated by dividing the number of copies in each fraction by the total number of copies (cyto + nuc). Data correspond to one RT experiment followed by PCR reactions performed in triplicates

The next step was to examine whether the two RT-qPCR protocols were indeed strand-specific. Non-specific priming during RT (mispriming) is a common phenomenon documented in various studies [32, 33]. We therefore assessed whether the PCR protocol from one orientation can or not amplified products from the other orientation. As a control for non-specific priming, a classical oligo-dT RT + qPCR was performed to amplify the RNA from EEF1G, a house-keeping gene that level of expression in T cells is moderated (EEF1G, GeneCards database), to match the level of HIV-1 RNAs. Consistent with non-specific priming, we found that the EEF1G RNA could not only be amplified upon oligodT RT but also upon a sense or antisense tagged-RT (Fig. 3A). In contrast, when either the sense or antisense tagged RT was used, the RT products could be only amplified by PCR primers specific to the same orientation (Fig. 3B), indicating a lack of mispriming. As a complementary validation approach, we took advantage of the reported distinct localizations of the sense usRNA (predominantly cytoplasmic) and antisense (predominantly nuclear) RNAs [20]. Cell fractionation experiments were conducted in two T cell lines latently infected with HIV-1: ACH-2 T cells that carry a complete provirus with mutations in the TAR element [34] and J1.1 T-cells that carry a complete but repressed wild-type provirus [35]. ACH-2 T cells have been shown to exhibit a Rev-deficient phenotype [36] that results in nuclear retention of the sense usRNA. In good agreement with this defect, we found that in ACH-2 cells, the sense RTqPCR detected RNA predominantly in the nuclear fraction (Fig. 3C, left panel). In contrast, in J1.1 T cells that do not exhibit the Rev-negative phenotype, the sense RTqPCR detected RNAs almost exclusively in the cytoplasmic fraction (Fig. 3C, left panel). With respect to antisense RTqPCR, amplified products were detected exclusively in the nuclear fraction in ACH-2 (98%) or predominantly (80%) in J1.1 (Fig. 3C, right panel), showing a distribution pattern clearly different from that of the products detected by the sense RTqPCR. Next, fractionation experiments were performed with productively-infected T cells, using Jurkat T cells infected with the NL4.3 virus for 24–48 h. The cellular distribution of the amplified products also clearly differed according to the RTqPCR protocol used, with sense RTqPCR detecting 50 to 60% of viral RNAs in the cytoplasmic fraction for 90% of the viral RNAs in the nuclear fraction for antisense RTqPCR (Fig. 3D). These data show that the two RTqPCR protocols prevent cross-detections and quantify distinct populations of HIV-1 RNAs, which validated their strand-specificities. They also confirm that the antisense HIV-1 RNAs are predominantly nuclear in both latently and productively-infected T cells.

### Quantification of the usRNA and antisense RNAs in primary infected CD4 + T cells

**Fig. 4:**
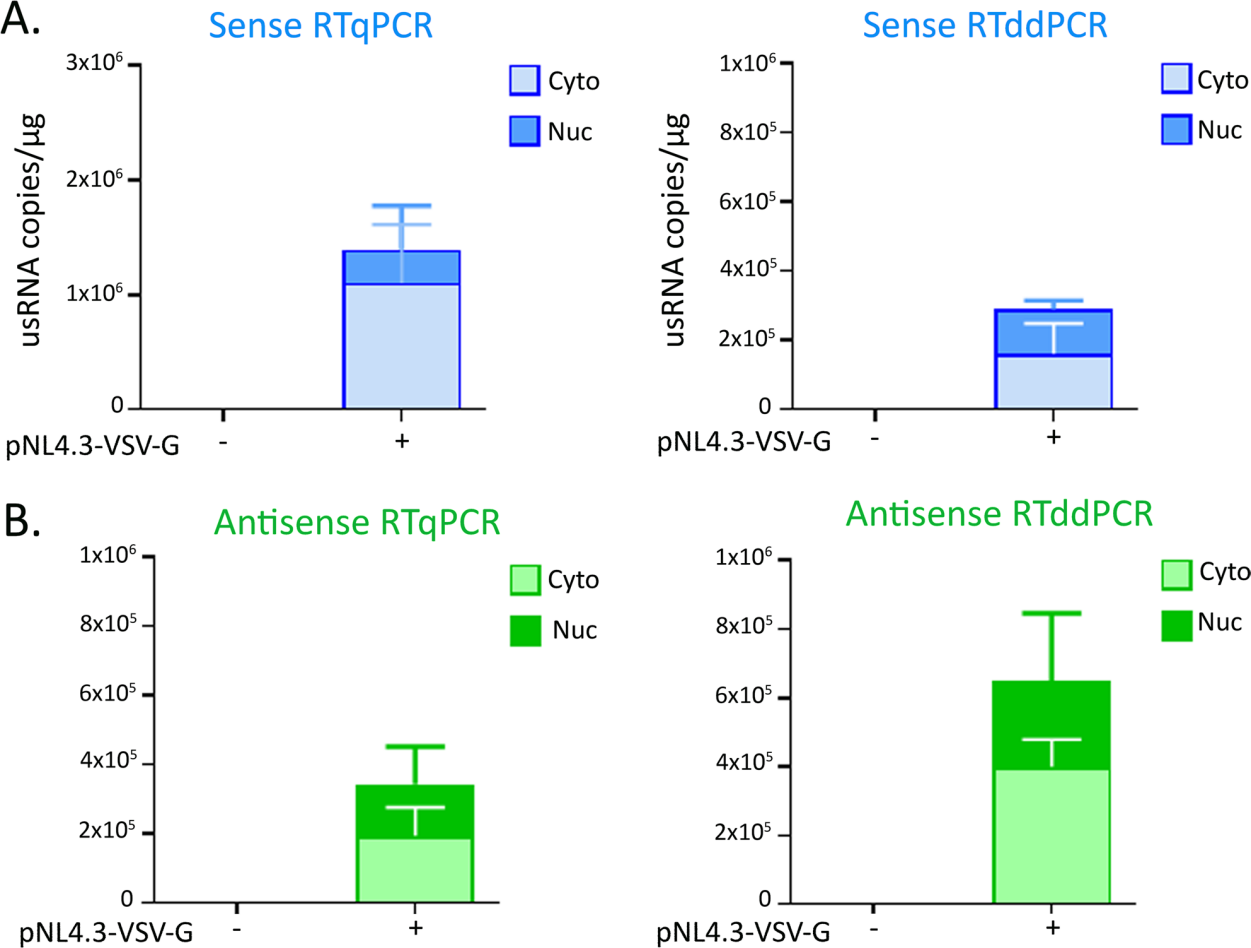
Comparison of RTqPCR and RTddPCR for the quantification of sense and antisense RNAs in primary CD4+ T cells.Experiments were performed using sorted primary CD4+ T cells obtained from one donor, cultivated in the presence of IL-7 (10 ng/mL) before and during infection with NL4.3-VSV-G viruses in three separate wells. Uninfected sorted primary CD4+ T cells were also analyzed as negative controls. Cell fractionations were performed 5-day post-infection to prepare cytoplasmic and nuclear RNAs. A. Quantifications of the sense usRNA or B. Quantifications of antisense RNAs were conducted by either RTqPCR or RTddPCR, as indicated. For RTqPCR, data correspond to one RT experiment per infection, followed by PCR performed in triplicates. For RTddPCR at least two independent RT reactions were performed by infection followed by ddPCR performed in duplicates or triplicates.

The quantification of cDNA produced from cell-associated HIV-1 RNAs classically conducted by qPCR, is increasingly performed via droplet digital PCR (ddPCR), a method believed to be more sensitive and repeatable than RTqPCR [28]. Therefore, we compared the results of quantification via either RTqPCR or RTddPCR using a relevant cell model, HIV-1-infected primary CD4 + T cells. To promote survival as well as increased susceptibility to HIV-1 infection, sorted CD4 + T cells were cultivated in the presence of a low IL-7 dose (10 ng/mL) before and during infection with NL4.3-VSV-G viruses. After infection was performed in three wells, cell fractionation was performed to quantify HIV-1 RNAs in the cytoplasmic or nuclear fraction. Because of greater variations in the number of copies obtained in each of the three infections, usRNA quantification was less accurate using RTqPCR than via RTddPCR (Fig. 4). However, the usRNA was predominantly found in the cytoplasmic fraction using each method (Fig. 4A). For the antisense RNAs, RTqPCR and RTddPCR provided similar data in terms of their cellular distribution. However, a greater number of copies were obtained by RTddPCR, suggesting that this method is more efficient for the quantification of antisense RNAs (Fig. 4B). This shows that the HIV-1 antisense RNAs produced in primary HIV-1 infected-CD4 + T cells can also be accurately quantified, with the best results obtained via RTddPCR.

### Expression of usRNA and AST in splenocytes and PBMCs from PLWH

**Fig. 5:**
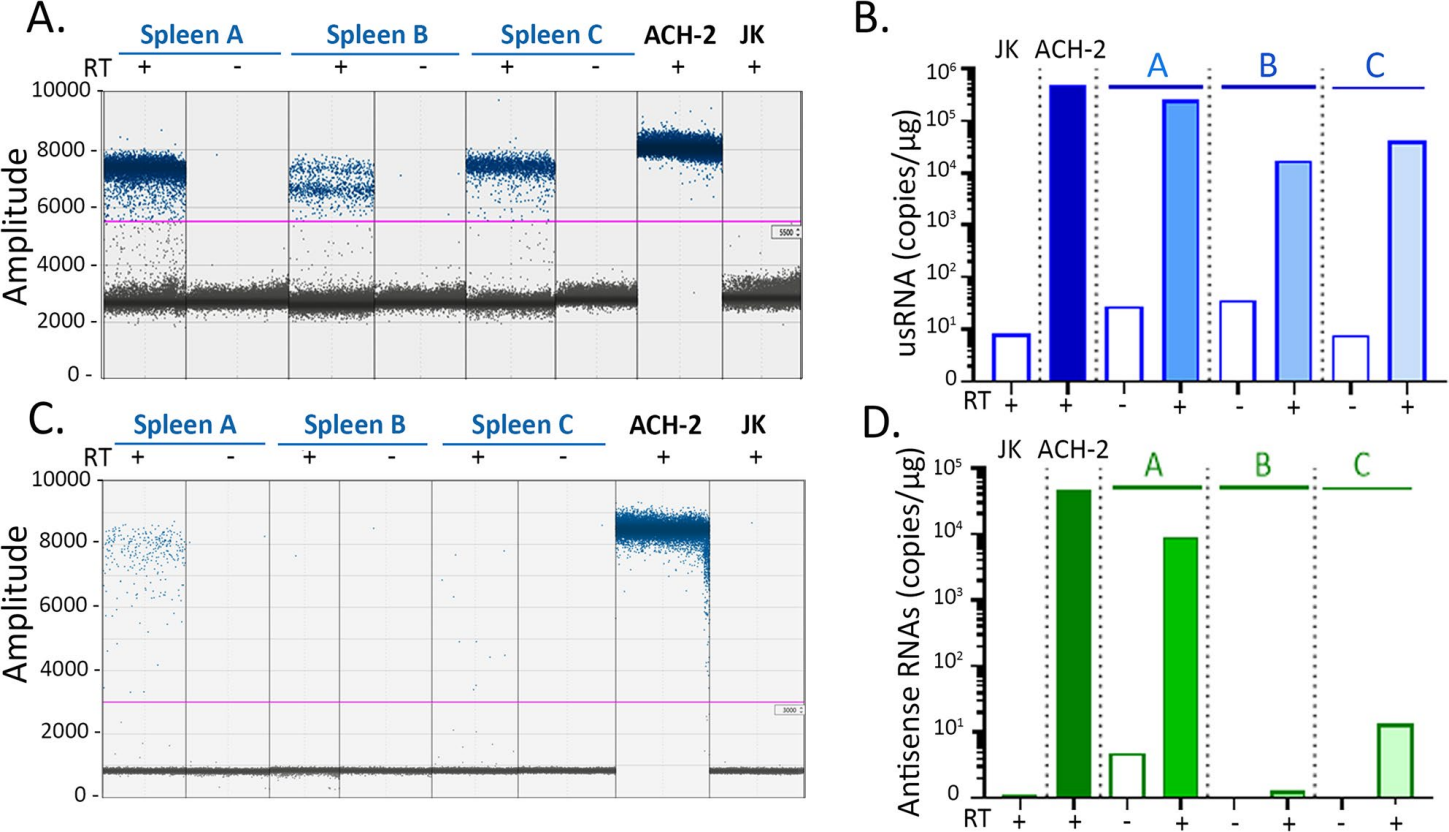
Quantification by RTddPCR of sense and antisense RNAs in spleen samples from untreated PLWH. Experiments were performed using cryopreserved total RNA extracted from spleen cells from three untreated PLWHs and ACH-2 or Jurkat T cells as controls. Quantifications of **A**, **B**. the sense usRNA or **C**, **D**. antisense RNAs conducted by RTddPCR. **A** and **C** Raw data obtained for one representative ddPCR quantification. The pink line represents the positivity threshold. **B** and **D** show the combined data of two independent ddPCR quantifications

Having demonstrated that our protocols can quantify HIV-1 sense and antisense RNA in HIV-1-infected primary T cells, we proceeded to study whether they could also be used to quantify these RNAs in PLWH. We chose to use the RTddPCR protocols validated above that appeared to be more efficient than RTqPCR. As proof of concept of virus detection in clinical samples, we first used spleen biopsies since splenocytes from untreated PLWH were shown to display a high frequency of HIV-1-infected CD4 + T cells [37, 38]. Experiments were done with cryopreserved total RNAs prepared from spleen biopsies of three untreated PLWHs (A, B and C) and with ACH-2 cells and Jurkat T cells as positive or negative control (Fig. 5). As only RNA samples were available, it was not possible to perform cell fractionation in these experiments. The data of one representative RTddPCR quantification indicated clear separation between negative and positive droplets for both usRNA and antisense RNA quantifications, validating the experiments. In addition, no positive droplet was visible in the negative control performed without RT or in Jurkat T cells (Fig. 5A and C). The usRNA was detected in the three spleen samples (Fig. 5B) with a number of copies ranging from 2 to 20 × 10^4^ copies/µg of total (Fig. 5B). We noticed that a dual positive population was observed for sample B, which in our experience depends on the quality of RNA preparations or RT reactions but did not invalidate the results (Fig. 5A). Antisense RNAs were detected in two out of the three samples, at a significant level in sample A (10^4^ copies/µg of total RNAs) and at low level in sample C (10 copies/µg of total RNAs) (Fig. 5D).

**Fig. 6:**
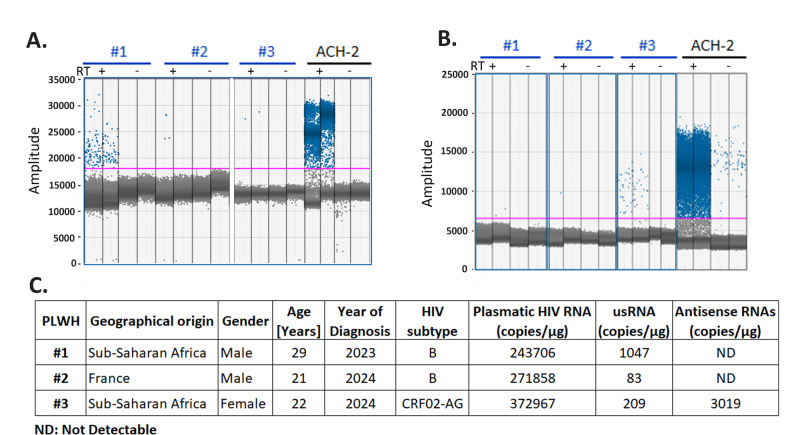
Quantification by RTddPCR of sense and antisense RNAs in PBMCs from untreated PLWH. Experiments were performed using total RNA extracted from cryopreserved PBMCs from three untreated PLWHs and ACH-2 as positive control. **A** Raw data obtained for the usRNA. **B** Raw data obtained for the antisense RNAs. For the sense RTddPCR, experiments were performed using 275 ng of total RNA for PBMC #1, 82 ng of total RNA for PBMC #2 and 20 ng of total RNA for PBMC #3 and for the antisense RTddPCR, experiments were performed using 275 ng of total RNA for PBMC #1, 31 ng of total RNA for PBMC #2 and 20 ng of total RNA for PBMC #3. The pink line represents the positivity threshold. **C** Virological parameters and numbers of copies for the sense and antisense RNAs in the three PBMC samples

We next assessed the efficiency of our protocols for detecting HIV-1 RNA in PBMC from PLWH, in which the frequency of CD4 + HIV-1 infected T cells is substantially lower in blood than in spleen [37, 38], increasing thereby the background due to cellular RNAs. PBMCs from three untreated PLWH were purified from blood samples and total RNAs were extracted without prior culture or stimulation. The usRNA was detected in the three PBMCs samples with values of 83, 209 and 1047 copies/µg of total RNA (Fig. 6A and C). In contrast, antisense RNAs were only detected in one sample with a number of copies of 3019 copies/µg of total RNA (Fig. 6B and C). Interestingly, a CFR02 subtype (Fig. 6C) was identified for this sample, showing that the protocols were also efficient to quantify sense and antisense HIV-1 RNA in a non-B PLWH. These data validate our protocols of RTddPCR for the quantification of the usRNA and antisense RNAs from total RNAs from spleen or PBMCs of PLWH with either B or CRF-02 subtypes.

## Discussion

Given that viral antisense products can modulate the HIV-1 life cycle, the level of HIV-1 antisense RNA relative to sense RNA appears to be an important indicator for monitoring viral replication, latency, and reactivation. The aim of this study was therefore to establish sensitive and strand-specific RT-PCR protocols for the comparative analysis of sense and antisense transcription that can be used in clinical studies.

To date, comparison of antisense and sense expression was done using the mono-spliced *env* transcript as sense RNA [24, 25]. However, the *env* transcript can rarely be detected in PLWH under ART, in contrast to the unspliced sense RNA (usRNA) [39]. We therefore decided to develop two strand-specific RTqPCRs methods to quantify the usRNA as a proxy of sense transcription, together with the antisense RNAs. Furthermore, to apply these protocols to PLWH, we considered the genetic diversity of the HIV-1 strains. Several strains of HIV-1 circulate worldwide, the HIV-1 B subtype being predominant in North America, the Caribbean and Western Europe [40, 41]. However, the frequency of non-B subtypes has increased over time in these countries. For instance, during the 2014–2016 period, CRF02 and other recombinants accounted for more than 40% of cases in France [42, 43]. For quantifying the usRNA, the conserved nuc 1280–1362 region positioned downstream the spliced donor site was chosen. For the antisense RNAs, we focused on the 7787–7869 part of the genome, in which we could select primer and probe sequences with high degrees of conservation within clade B as well as CRF-02 and CRF-06 recombinant strains.

The subsequent crucial step was to confirm that the two protocols were indeed strand-specific. We first provided evidence that cDNA generated using the tagged RT-primer specific for one orientation could not be quantified by PCR performed with primers specific to the opposite orientation, excluding the presence of cross-contamination between sense and antisense RNAs during the PCR steps. Furthermore, cell fractionation revealed that the HIV-1 RNAs detected by the sense RTqPCR in both the latent T-cell J.1.1 and acutely-infected Jurkat T cells exhibited the phenotype of the usRNA, i.e. cytoplasmic localization. Conversely, in the latent T-cell line ACH-2, which harbors a mutation in the TAR element that induces a Rev-defective phenotype, the sense RT-qPCR detected, as expected, RNAs in the nucleus. In contrast, the antisense RT-qPCR predominantly quantified RNAs within the nuclear fraction in the two latently infected T-cells, as well as in the productively-infected Jurkat T cells. These findings validated the strand-specificities of the two RT-qPCRs methods and confirmed the nuclear localization of antisense RNAs in latently and acutely infected T cells.

Experiments with primary CD4 + T-cells also enabled comparisons of quantification methods, RT-qPCR and RT-ddPCR. Quantification by qPCR necessitates the generation of a standard curve for the calculation of copy number, whereas ddPCR directly determines the absolute number of copies. For usRNA, greater variation between infections was detected *via* RTqPCR than *via* RTddPCR and for antisense RNAs, more copies were detected *via* RTddPCR than *via* RTqPCR. We then concluded that RTddPCR would be the best method for quantifying HIV-1 RNAs in clinical samples. Interestingly, the two approaches revealed a significant number of antisense RNA copies in the cytoplasm of primary infected CD4 + T cells, which differed from the findings in latent T cells and acutely-infected Jurkat T cells. This may suggest that the cellular localization of antisense RNAs may be subject to changes depending on the cellular context. Given that IL-7 induces CD4 + T cell survival and stimulates HIV-1 replication [44], it could also be envisioned that IL-7-induced cellular proteins and/or HIV-1 sense proteins may trigger the cytoplasmic export of the antisense RNAs. It would be interesting to examine whether such an increase in cytoplasmic antisense RNAs coincides with increased production of the antisense protein ASP, which might thereby exert a positive influence on viral replication. Unfortunately, we were unable to detect ASP in infected T-cells, which is consistent with existing data reporting that ASP detection requires optimized expression vectors [21].

The final objective of our study was to determine whether our RT-PCR protocols were sensitive enough to detect HIV-1 RNAs in samples obtained from PLWH. Indeed, we showed that our protocols were able to detect usRNAs in three spleen samples and three PBMC samples from untreated PLWH, and antisense RNAs in two out of three spleen samples and one out of three PBMC samples form untreated PLWH. That antisense RNAs were not detected in samples in which significant amounts of the usRNA were detected provided definitive evidence that contamination of the antisense RNAs by sense RNAs did not occur during the PCR. In spleen sample A, the level of antisense RNAs reached approximately 10% of the level of the usRNA and in PBMCs sample #3, the number of copies of antisense RNAs was 14-fold higher than the level of usRNA. This suggests that antisense transcription can be significant, even predominant, in tissue and blood during untreated infection. Recent data published by Capoferri et al. also described production of HIV-1 antisense RNAs in vivo. They reported that the level of antisense RNA was lower in untreated PLWH than in PLWH, with in mean < 2 and 26 copies/100 infected PBMC, respectively [25]. Since in our case, we do not know the number of infected PBMC in the samples we tested, it is difficult to directly compare these data to our results. However, our data obtained from spleen sample A and PBMC #3 showed that, in the absence of treatment, antisense RNAs can be expressed at a high level in tissue or blood. Interestingly, we found that compared to latent cells, antisense RNAs are enriched in the cytoplasm in productively infected primary CD4 + T cells. Such localization could favor the expression of the ASP protein over that of repressive antisense RNAs, facilitating thereby virus propagation among CD4 + T cells [10]. In this sense, ASP represents a novel interesting target for therapeutic strategies aiming at blocking HIV replication in vivo.

As only three spleen samples and three PBMC samples from untreated PLWHs were tested, it is difficult to draw any conclusions regarding the respective levels of expression of sense and antisense RNAs during acute infection in vivo. However, no correlation was found between the levels of sense and antisense RNAs, nor between plasmatic viral RNA levels and either sense or antisense RNA levels. This suggests that sense and antisense transcriptions may be regulated by distinct viral or cellular mechanisms.

As mentioned earlier, the previous studies describing antisense RNA expression in vivo were developed for subtype B strains. Our analysis of virus variability showed that the primers and probes used in these studies will not be optimal to detect non-B viruses, while we showed that our primer and probe sequences are also conserved in CRF-02 subtypes. Consistently, expression of antisense RNAs was detected in PBMCs from sample #3, exhibiting a CRF-02AG viral subtype. The fact that our primers and probe sequences are also conserved in CRF-06 subtype, suggests that antisense RNAs from this subtype would also be detected with our protocols.

## Conclusions

This study demonstrates that HIV-1 antisense RNAs are expressed in the spleen and PBMC of a proportion of PLWH. Further studies involving a larger number of PLWH are necessary to determine the frequency of antisense transcription in vivo, in relation to the immune-virological parameters of the PLWH. Additionally, given the potential of non-coding antisense RNAs to induce and reinforce HIV-1 latency, it would be interesting to quantify their expression in long-term non-progressors and post-treatment controllers. Monitoring the sense/antisense ratio in situations of viral rebound due to treatment interruption would also be very informative. Such studies would facilitate a more comprehensive understanding of the dynamics of HIV-1 sense and antisense transcript expression in vivo and could pave the way for novel strategies to control HIV-1 infection.

## Supplementary Information

Below is the link to the electronic supplementary material.

Supplementary Material 1.

## Data Availability

No datasets were generated or analysed during the current study.
