# Supplementary material for: Strand-specific detection of cell-associated sense and antisense HIV-1 RNAs in splenocytes and PBMC from PLWH
Source: Retrovirology. 2025 Dec 24;23:2. doi: 10.1186/s12977-025-00670-5 (PMC12849480; doi:10.1186/s12977-025-00670-5)
Supplement: Supplementary file 1 — Supplementary Material 1. [file 12977_2025_670_MOESM1_ESM.docx]

**Supplemental Figure S1**

**A.**

***** Sequence also used in Capoferri *et al*., 2025 [24]

**B.**

**Fig. S1:** **Analysis of conservation among the B and CRF02/06 subtypes of primers and probes used in previous studies**. The conservation of each primer and probe position used in (**A**) Zapata *et al*. 2017 [15] and (**B**) Kobayashi-Ishihara *et al*. 2012 [17] was assessed using the 2021 HIV Compendium among 1,504 B and 133 CRF-02+CRF-06 sequences. Positions with a degree of variability lower than 80% are indicated in red.

**
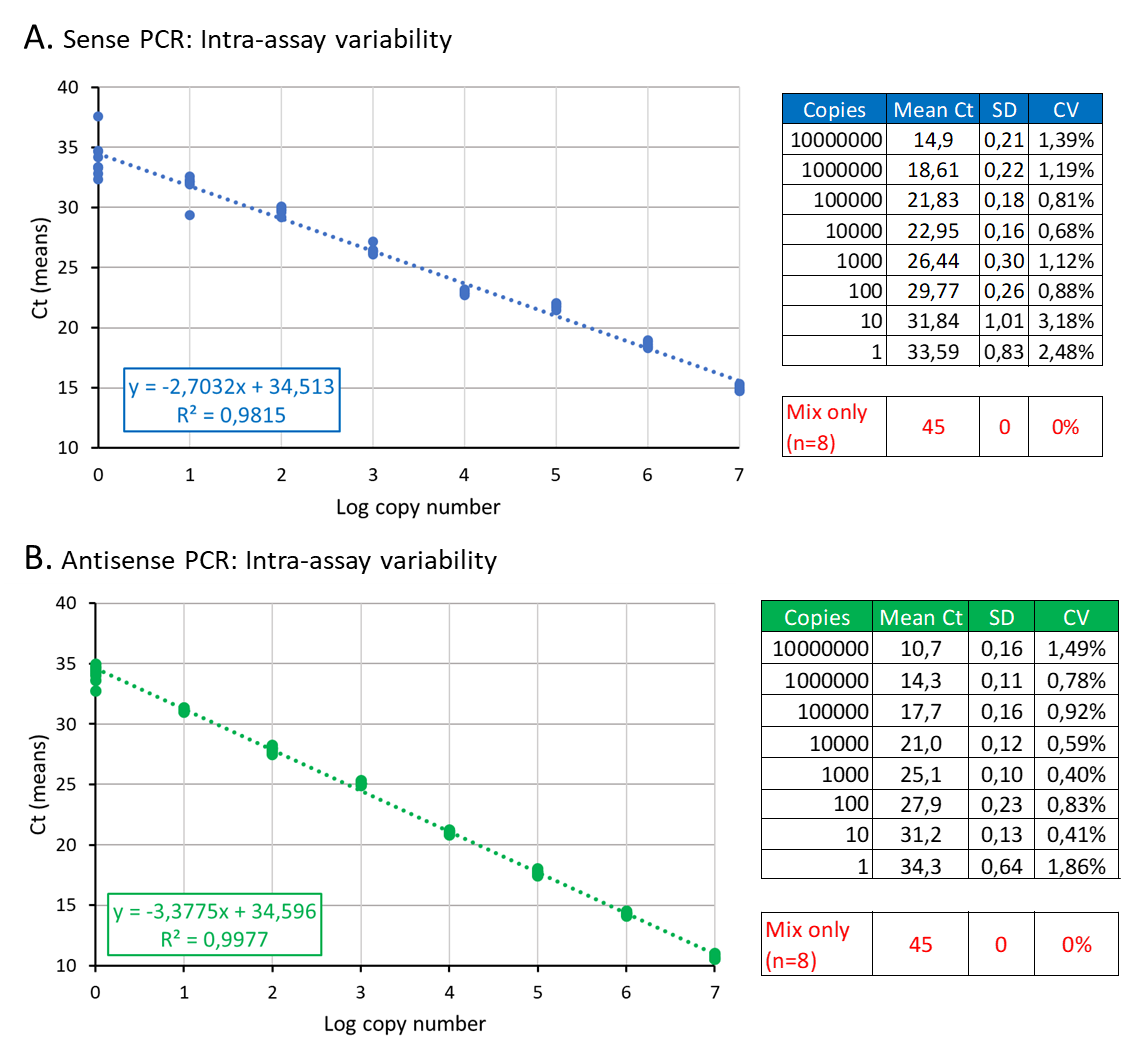
Supplemental Figure S2**

**Fig. S2: Intra-assay variability of the two RTqPCR.**

Intra-assay variability was assessed by 10 parallel quantifications in duplicates of the same dilutions of the dsDNA corresponding to the sense or antisense amplicons. The right tables show the mean Ct values as well as the standard deviation and coefficients of variation for each number of copies.
